# The Microbiome: A New Target for Research and Treatment of Schizophrenia and its Resistant Presentations? A Systematic Literature Search and Review

**DOI:** 10.3389/fphar.2018.01040

**Published:** 2018-10-15

**Authors:** Alessandro Cuomo, Giuseppe Maina, Gianluca Rosso, Bruno Beccarini Crescenzi, Simone Bolognesi, Angela Di Muro, Nicola Giordano, Arianna Goracci, Stephen M. Neal, Maria Nitti, Fulvio Pieraccini, Andrea Fagiolini

**Affiliations:** ^1^Department of Molecular and Developmental Medicine, University of Siena, Siena, Italy; ^2^Department of Neuroscience, University of Torino, Turin, Italy; ^3^Psychiatric Unit, San Luigi Gonzaga Hospital of Orbassano, University of Torino, Turin, Italy; ^4^Department of Medicine, Surgical and Neurological Sciences, University of Siena, Siena, Italy; ^5^Department of Psychiatry, West Virginia School of Osteopathic Medicine, Lewisburg, WV, United States

**Keywords:** microbiome, schizophrenia, resistant, inflammation, probiotics, gut, immune, interleukin

## Abstract

**Background:** The gastrointestinal system hosts roughly 1,800 distinct phyla and about 40,000 bacterial classes, which are known as microbiota, and which are able to influence the brain. For instance, microbiota can also influence the immune response through the activation of the immune system or through the release of mediators that are able to cross the brain blood barrier or that can interact with other substances that have free access to the brain, such as tryptophan and kynurenic acid, which is a metabolite of tryptophan and which has been involved in the pathogenesis of schizophrenia.

**Objectives:** This paper reviews the possible relationships between microbiome, schizophrenia and treatment resistance. Given the possibility of a role of immune activation and alterations, we also describe the relationship between schizophrenia and immune inflammatory response. Finally, we report on the studies about the use of probiotic and prebiotics in schizophrenia.

**Methods:** Cochrane library and PubMed were searched from the year 2000 to 2018 for publications about microbiome, immune-mediated pathology, schizophrenia and neurodevelopmental disorders. The following search string was used: (microbiome or immune mediated) AND (schizophrenia OR neurodevelopmental disorder). Associated publications were hand-searched from the list of references of the identified papers. A narrative review was also conducted about the use of probiotics and prebiotics in schizophrenia.

**Results:** There exists a close relationship between the central nervous system and the gastrointestinal tract, which makes it likely that there is a relationship between schizophrenia, including its resistant forms, and microbiota. This paper provides a summary of the most important studies that we identified on the topic.

**Conclusions:** Schizophrenia in particular, remain a challenge for researchers and practitioners and the possibility of a role of the microbiome and of immune-mediated pathology should be better explored, not only in animal models but also in clinical trials of agents that are able to alter gut microbiota and possibly influence the mechanisms of gastrointestinal inflammation. Microbiome targeted treatments have not been well-studied yet in patients with mental illness in general, and with schizophrenia in particular. Nonetheless, the field is well worth of being appropriately investigated.

## Introduction

Human body is colonized by various bacteria and the majority of them are within intestines, ranging from <105 bacteria per gram of digesta in the upper parts of the small intestine, to >1,012 bacteria per gram of digesta in the large intestine (15). Indeed, the gastrointestinal system hosts roughly 1,800 distinct phyla and about 40,000 bacterial classes, which are known as microbiota (Sherwin et al., 2016).

Microbiota main component include the following phyla: Firmicutes, Bacteroidetes, Proteobacteria, Actino-bacteria, Fusobacteria, and Verrucomicrobia. Those microorganisms play important role in maintaining homeostasis and their imbalance may lead to various diseases (Eckburg et al., 2005). Newer research has made it clear that the maintenance of gut homeostasis is important for the prevention and treatment of various neurological diseases, possibly including schizophrenia (Galland, 2014; Severance et al., 2015a). The link between the gastrointestinal system and the brain is bidirectional and it is performed through several pathways. As the brain might affect functioning of the gut, modify microbial habitat and hence influence the microbiota composition (Bruce-Keller et al., 2018), at the same time, any disturbance of the microbial flora on the surface of intestinal mucosa might lead to a number of neuropsychiatric conditions, including schizophrenia (Petra et al., 2015; Zhu et al., 2017). Based on the above observations, the relationship between the brain and the gut has become a target for the research on the pathogenesis and treatment of several illnesses, including schizophrenia. Although collecting and storing stool samples from individuals with schizophrenia is challenging, interesting results have been found though the study of the oropharyngeal microbiome (Castro-Nallar et al., 2015; Bruce-Keller et al., 2018).

Alterations in the microbiota composition might contribute to symptoms of schizophrenia through an immune response or through the release of mediators, such as, amino acids, that are able to cross the brain blood barrier or that are able to interact with other substances that have free access to the brain. For instance, it is well-known that the microbiota can influence plasma level and metabolism of tryptophan and kynurenic acid, which is a metabolite of tryptophan (Bruce-Keller et al., 2018; Gao et al., 2018). This influence has been suggested as a possible contributor to the pathogenesis of schizophrenia. Of interest, kynurenic acid is a NMDA receptor antagonist which has been involved in the pathogenesis of schizophrenia, thus confirming the possibility of the gut to influence the brain (Erhardt et al., 2003; Balu, 2016). Also, microbiota can change host physiology through the production of metabolites such as, 5-hydroxytryptophan and γ-aminobutyric acid (GABA). For instance, Bifidobacteria and Lactobacilli can generate of GABA, the Bacillus family can generate dopamine and noradrenaline, and Escherichia can generate noradrenalin and 5-HT. In addition, germs like Clostridium sporogenes decarboxylate tryptophan to tryptamine, preventing the absorption of this essential amino acid (Wall et al., 2014; O'Mahony et al., 2015).

Converging lines of evidence indicate a link between schizophrenia and immune activation (Dickerson et al., 2017; van Kesteren et al., 2017), a hypothesis that dates back to the 1950s (Severance et al., 2012). Alterations of the immune system have been described in individuals with schizophrenia, including T-cell activation, increased plasma cytokines, chemokines, and increased acute phase reactants (Kronfol and Remick, 2000; Bruce-Keller et al., 2018). Of interest, antipsychotic medications may exert anti-inflammatory effects (Maier et al., 2018) and non-steroid anti-inflammatory agents can reduce symptom severity in patients with an altered immune response (Zheng et al., 2017).

This paper reviews the possible roles of the microbiome in the development and evolution of schizophrenia, with the ultimate goal to explore the possibility of developing new treatments, especially for those patients with treatment resistant presentations. Although studies on the fecal microbiome in schizophrenia and large multi-sites placebo-controlled trials of medications able to modify gut microbiota or inflammation in patients with schizophrenia are still lacking, this line of research is exciting and may hold promise.

## Methods

### Searches

Cochrane library and PubMed were searched from the year 2000 to 2018 for publications about microbiome, immune-mediated pathology, schizophrenia and neurodevelopmental disorders. The following search string was used: (microbiome or immune mediated) AND (schizophrenia OR neurodevelopmental disorder). Associated publications were hand-searched from the list of references of the identified papers. We included “neurodevelopmental disorder” in our preliminary search string despite the recent challenges to the neurodevelopmental hypothesis of schizophrenia, given that neurodevelopmental abnormalities may nonetheless contribute to- or share pathophysiological mechanism with- schizophrenia (Owen et al., 2011). Registries of clinical trials in controlled trials.com and clinical trial.gov were also scrutinized.

### Study selection

**Figure 1:**
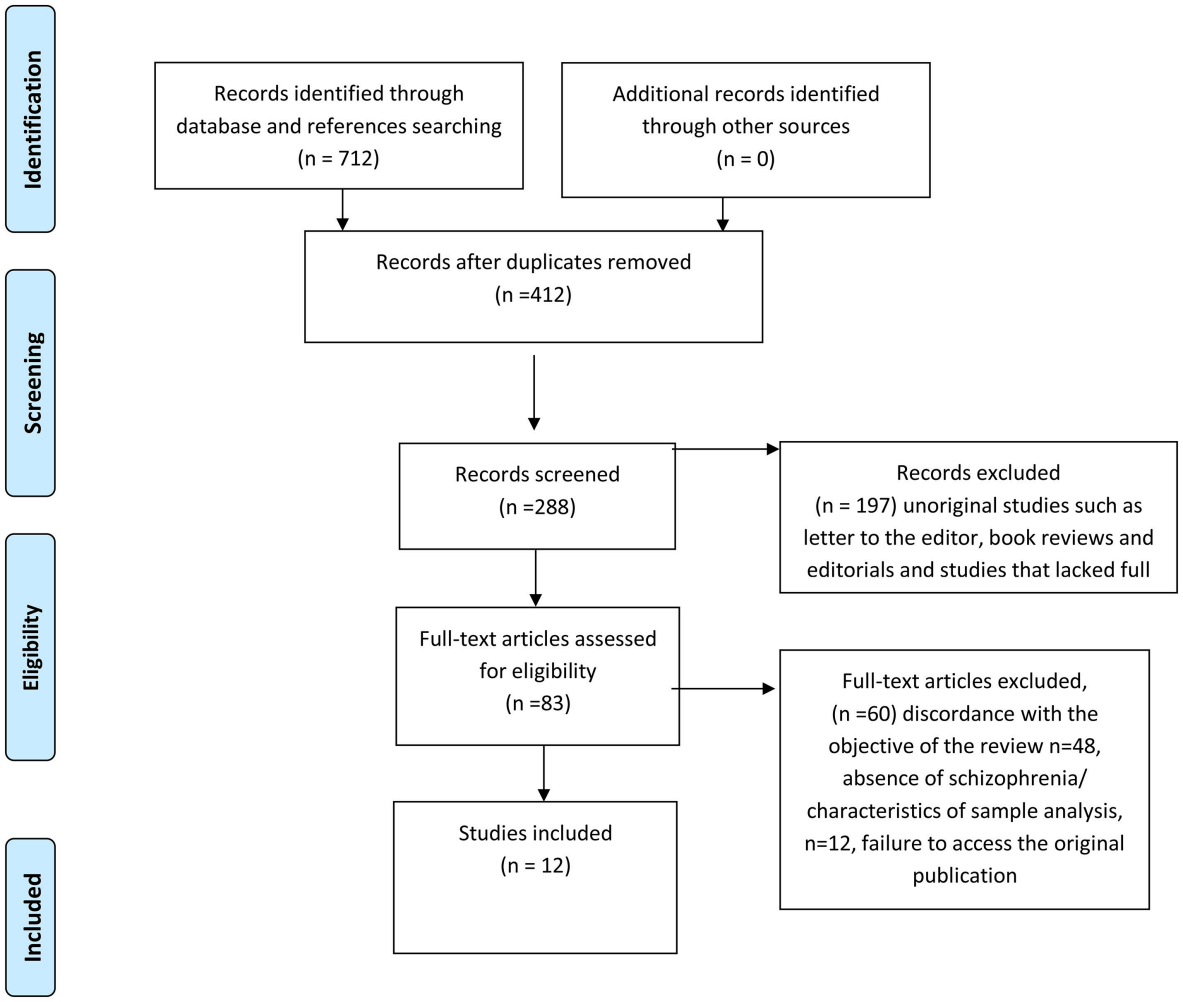
Prisma Flow Diagram.

We used the Preferred Reporting Items for Systematic Reviews and Meta-Analyses (PRISMA) course of action (Figure 1). We deliberately chose a broad search string, given that the research on the relationship between schizophrenia and microbiome is still in its infancy and that we wanted to search as many papers as possible. However, the main criteria to select the articles for this review were the following: the study pertained to microbiome; a schizophrenia subgroup (or sample) was part of the study; the study outcomes for the schizophrenia subgroup (or sample) were presented. Hence, the majority of selected papers resulted from studies pertaining to the relationship between microbiome, schizophrenia and the inflammatory-immunity response system. Although the papers pertaining to review studies, animal research, non-original studies such as, letters to the editor, book reviews and editorials, were excluded from the in-depth analysis, they were still considered for our preliminary studies and discussion, and quoted as necessary. As a second step, we conducted a narrative review about the use of probiotics and prebiotics in schizophrenia.

### Quality assessment

First-grade studies included research on schizophrenic's microbiota. The second grade included studies of gastrointestinal inflammation, infection and antimicrobial drugs in schizophrenia that could be related to microbiome.

## Results

This section reports the most relevant studies pertaining to: (1) the relationship between microbiome and schizophrenia; (2) the relationship between schizophrenia and immune inflammatory response; and (3) studies about probiotic and prebiotics, with special reference to schizophrenia.

### Relationship between microbiome and schizophrenia

Castro-Nallar and colleagues compared the oropharyngeal microbiome of schizophrenic patients and healthy subjects and found that certain types of bacteria were significantly predominant in patients suffering of this mental condition. In addition, they observed differences in the amount and distribution of species, as well as different metabolic pathways. In schizophrenic patients lactic acid bacteria and metabolite transport system, respectively, were predominant (Castro-Nallar et al., 2015). Yuan et al. (2018) evaluated the alterations in microbiota in 41 drug naïve, first episode patients with schizophrenia, after 24-weeks of treatment with risperidone. Compared to healthy controls, schizophrenic patients had significantly lower number of fecal Bifidobacterium, Escherichia coli and Lactobacillus. Conversely, they had significantly higher number of fecal Clostridium coccoides. After 24-weeks of treatment with risperidone, a significant increase in the numbers of fecal Bifidobacterium and E. coli was observed. Also, the patients showed a significant decrease in the number of fecal Clostridium coccoides and Lactobacillus. The authors concluded that drug naïve, first episode patients with schizophrenia show abnormalities in microbiota composition and observed that risperidone treatment caused significant changes in fecal bacteria. Also, they hypothesized that those changes were associated with the metabolic changes induced by risperidone.

Schwarz et al. (2018) evaluated the differences in fecal microbiota between 28 individuals diagnosed with first-episode psychosis (half of whom received a diagnosis of schizophrenia by the study 1-year follow up assessment) and 16 matched health controls. They found that psychotic patients had higher number of Lactobacillus bacteria. Of interest, the subgroup of patients that showed the strongest differences in microbiota coincided with the subjects who had a poorer response after up to 12 months of treatment.

Shen et al. (2018) evaluated the difference in gut microbiota between 64 patients with schizophrenia and 53 healthy controls and found a higher number of Proteobacteria, Succinivibrio, Megasphaera, Collinsella, Clostridium, Klebsiella and Methanbrevibacter and a lower number of Blautia, Coprococcus, Roseburia in patients with schizophrenia compared to healthy controls. Interestingly, the authors observed that 12 microbiota could be used as diagnostic factors to distinguish the patients with schizophrenia from the control cohort. Yolken et al. (2015) investigated the relationship between schizophrenia and bacteriophage genomes. They enrolled 41 persons with schizophrenia and 33 healthy controls. They came to conclusion that Lactobacillus phage phiadh was significantly more prevalent in the oropharynx of patients with schizophrenia. The presence of this microorganism also correlated with immunological disorders and valproate administration in the study group.

Severance et al. (2013) measured serological surrogate markers of bacterial translocation [soluble CD14 (sCD14) and lipopolysaccharide binding protein (LBP)] in two cohorts. The first one included 141 patients suffering from schizophrenia, 75 with bipolar disorder and 78 controls. In the other cohort there were 78 with antipsychotic naïve first-episode schizophrenia and 38 with medicated first-episode schizophrenia. They found that soluble CD14 was more prevalent in patients with schizophrenia and both sCD14 and LBP correlated with CRP in that group. Critchley and Harrison (2013) evaluated the impact of visceral homeostasis on both physiological and mental capacities of the brain and added to the studies showing that microbiota might affect gut-brain axis at any age, leading to neurodevelopmental or neurodegenerative conditions (Dinan and Cryan, 2017).

### Relationship between schizophrenia and immune inflammatory response

Several studies have pointed to an association between schizophrenia the immune-inflammatory response system (IRS), For instance, it has been observed that people living with schizophrenia had more prevalent inflammatory cytokine L1 receptor antagonists (Severance et al., 2012). Also, Chengappa et al. (1995) showed that Caucasian patients suffering from schizophrenia with HLA B8/DR3 had impaired proliferative lymphocyte response. Ganguli et al. (1994) discovered elevated IL-6 levels in patients with schizophrenia. Maes et al. (1997a,b) wrote about immunological response in schizophrenia. Arolt et al. (1997) and Rothermundt et al. (1998) published similar findings about the correlation of schizophrenia with abnormal immunological response.

In a study involving 17 individuals with treatment-resistant schizophrenia (TRS) 14 patients with schizophrenia showing response to antipsychotic treatment and seven normal controls, IL-6 was significantly higher in individuals with schizophrenia than in healthy volunteers (HV), IL-1RA was significantly higher in the TRS individuals than in HV, whereas schizophrenic patients who were not treatment resistant showed intermediate values. The authors concluded that schizophrenia in general, and TRS in particular, are characterized by cell-mediated immunity activation, primarily in the monocytic arm (Maes et al., 2000).

Lin et al. (1998) examined serum Clara Cell Protein (CC16), an endogenous protein with anti-inflammatory and immunosuppressive properties, interleukin-6 (IL-6), IL-6 receptor (IL-6R), and IL-1R antagonist (IL-1RA) in subjects with schizophrenia and normal controls. IL-6 and IL-6R were significantly higher in patients with schizophrenia than in normal controls and IL-6 was significantly higher in TRS than in normal controls, whereas patients with non-resistant schizophrenia TRS had intermediate values. Serum CC16 was significantly higher in normal volunteers and schizophrenic patients without a positive family history than in schizophrenic patients with a family history for psychoses. A significant inverse relationship was found between CC16 and IL-6 and IL-6R in patients with schizophrenia, but not in normal volunteers, once again pointing to the role of inflammation in schizophrenia, as indicated by higher IL-6 and IL-6R serum level, which may be linked to lower serum CC16. Müller et al. (1993) studied 55 patients with schizophrenia and observed a relationship between cellular immune parameters and the course of the psychopathological symptoms. It remains to be established what the cause is for IRS activation. IRS activation could be due to autoimmune responses (DeLisi et al., 1985; Margutti et al., 2006) or a microbial (i.e., viral) infection or reactivation (DeLisi and Crow, 1986). Kelly et al. (2017) suggested a strong role of the immune system in the development of schizophrenia; at the gene level, there is evidence about B-lymphocyte lineages that are included in the acquired immunity and major histocompatibility complex being related to this condition. Findings of schizophrenic patients with elevated levels of peripheral cytokines further confirm the possibility of a primary role of inflammatory processes. Benros et al. (2011) investigated if autoimmune diseases combined with exposures to severe infections may increase the risk of schizophrenia. They analyzed data from nationwide population-based registers in Denmark for the period from 1977 to 2006 and linked persons with autoimmune diseases and infections with individuals with diagnosis of a schizophrenia spectrum disorder in the Danish Psychiatric Central. They concluded that autoimmune disease might increase the risk of schizophrenia by 29%, and 60% in case of infection.

Dickerson et al. (2014) recently reviewed the literature concerning the immunity system, schizophrenia and bipolar disease and focused on two studies reporting differences in the oro-pharyngeal microbiota between schizophrenia cases and controls. The authors also pointed to studies showing higher rates of GI inflammation in patients with schizophrenia or bipolar disorder and on investigations focused on the relationship between psychiatric disorders and increased use of antibiotics, possibly mediated by antibiotic induced changes in microbiome.

Borovčanin et al. (2012) analyzed levels of serum type-1 cytokines, type-2 cytokines, type-17 cytokines and regulatory cytokines in 88 drug-naïve patients with first episode psychosis, 45 patients with schizophrenia in relapse and in the control group of 36 healthy persons. Patients with schizophrenia had elevated levels of IL-4 and increased production of TGF-β, which suggested the link between this psychiatric condition and chronic inflammatory processes. It is noteworthy that autoimmune conditions such as, celiac disease, i.e., a condition resulting from the interaction between certain dietary components and altered structure of the gastrointestinal tract, may be linked to schizophrenia. This link was first observed in epidemiological studies, and followed by research about common HLA predisposition (Severance et al., 2016). Efforts have been made to identify the role of the certain foods and inflammatory processes in bowels, and thus severity of symptoms of schizophrenia (Kraft and Westman, 2009).

### Studies about probiotic and prebiotics, with special reference to schizophrenia

In light of recent findings about the link between gut microbiota and schizophrenia, and the role of environmental factors in the development of this illness, there might be a possibility of using probiotics (containing species just as Lactobacillus and Bifidobacteria) in treatment of inflammatory processes within the gastrointestinal system, with positive effects of the symptoms of schizophrenia. Such treatment with “psychobiotics” could become a breakthrough in the management of mental illnesses (Saulnier et al., 2013; Sarkar et al., 2016; Deans, 2017). Severance and associates explored the possible relationship between food antigen-associated immune activation in patients with schizophrenia and gastrointestinal inflammation. They enrolled 193 subjects with non-recent and 67 with recent onset of schizophrenia, while there were 207 persons in the control group. They revealed food antigen antibodies and gastrointestinal inflammation in both schizophrenia groups (Severance et al., 2012).

In a study published in 2015, the same authors explored the link between dietary agents (wheat gluten and bovine milk casein) and immune response in blood and CSF samples in 105 patients with first episode of schizophrenia and 61 persons in the control group. In the experimental group IgG as a response to dietary proteins were significantly higher in both serum and CSF (Severance et al., 2015b).

Preliminary yet interesting information is emerging from clinical trials with probiotics in the treatment of schizophrenia (Bruce-Keller et al., 2018). Microbiome transplants from donor mice fed with high-fat diet showed that high fat-shaped microbiota disrupted cognitive, exploratory, and stereotypical/impulsive behaviors (Bruce-Keller et al., 2015). Other studies involving animal models demonstrated that probiotics may improve cognition, mood, anxiety, while improving neural activity and signaling (Sudo et al., 2004; Desbonnet et al., 2010; Bravo et al., 2011; Smith et al., 2014; Bruce-Keller et al., 2018). Also, mice studies have shown the ability of probiotics to promote hypothalamic synaptic plasticity and prevent decreases in hippocampal neurogenesis induced by stress (Ait-Belgnaoui et al., 2014). Dietary trans and saturated fats, may increase intestinal inflammation (Deopurkar et al., 2010; Okada et al., 2013), which results in a decrease of commensal Bacteroidetes and increase of pathogenic Enterobacteriaceae and Proteobacteria (Lupp et al., 2007; Stecher et al., 2007; Pédron and Sansonetti, 2008).

Karakuła-Juchnowicz et al. (2016) reviewed the role of the food antigens in schizophrenia, the use of diet modification, as well as antibiotics and probiotics as the possible treatment solutions. Probiotics are microorganisms, usually Lactobacilli and/or Bifidobacteria (Messaoudi, 2011; Tillisch et al., 2013; Steenbergen, 2015; Sarkar et al., 2016). Prebiotics are non-digestible carbohydrates that increase beneficial microbiota. Prebiotics may improve emotional affect and modulate stress responses (Schmidt et al., 2015). Randomized trials have shown efficacy of probiotics on mood (Messaoudi, 2011; Steenbergen, 2015) as well as the ability to reduce responses to stress (Kato-Kataoka, 2016). However, other studies have produced controversial results and therefore more trials are needed to completely demonstrate efficacy, to identify the specific strains that are most beneficial, as well as the correct dose and treatment duration (Doron and Snydman, 2015; Bruce-Keller et al., 2018). Similarly, large, controlled and well-powered studies about the efficacy of prebiotics are warranted (Bruce-Keller et al., 2018).

Tomasik et al. studied probiotic in schizophrenia and found their significant impact in reducing von Willebrand factor and increasing brain-derived neurotrophic factor (BDNF), monocyte chemotactic protein-1 (MCP-1), T-cell-specific protein RANTES, and macrophage inflammatory protein-1 beta (MIP-1) beta. Also, they found that that probiotic were related to regulation of intestinal immune and epithelial cells and suggested that supplementation of probiotics may improve control of gastrointestinal leakage in patients with schizophrenia (Tomasik et al., 2015).

Dickerson et al. (2014) performed a randomized, double-blind, placebo-controlled study and enrolled 65 patients with schizophrenia who were first treated with double blind probiotic or placebo for 14 weeks. Although no significant differences between probiotics and placebo groups were found in terms of changing schizophrenia symptoms severity, probiotics reduced the likelihood to develop severe bowel difficulty over the course of the trial.

Severance et al. (2017) conducted a randomized, placebo-controlled, longitudinal pilot study and explored the use of probiotics in treatment of both yeast gut infection and psychiatric symptoms 56 patients with schizophrenia. Probiotics were associated with a decrease of Candida albicans antibody levels as well as a decrease in gastrointestinal symptoms in male subjects and a trends for improvement in positive schizophrenia symptoms in males who received probiotics and were seronegative for C. albicans.

## Discussion

We reviewed the relationship between microbiome and schizophrenia as a means to speculate on the possibility that microbiome alterations play a role in schizophrenia in general, and in treatment resistant schizophrenia in particular. This field of investigation is in its initial phase but the potential role of the trillions of viruses, bacteria, and fungi that inhabit most of our body, from the gastrointestinal tract to the upper and lower airways, oral cavity, skin, urogenital tract, and even tissues once thought to be sterile, such as, blood or the eyes, is likely and worth being further explored. For instance, Castro-Nallar, Shen and Yolken's studies showed clear differences in the amount and/or distribution of germs species, as well as different metabolic pathways. However, only few studies have explored the relationship between microbiota and schizophrenia and—to our knowledge—no study has evaluated the relationship between microbiota and treatment resistance in schizophrenia. However, a hint came from Schwarz and colleagues' study. In fact, these authors correlated the numbers of Lactobacilli with severity along different symptom domains and observed that patients with the strongest microbiota differences were also those who showed poorer response to treatment.

We believe that the scarce data that is already available may be helpful to formulate new hypotheses and to stimulate further research toward a better understanding of the contribution of microbiome and immune-mediated abnormalities to schizophrenia and treatment resistant schizophrenia.

Although the study of microbiomes is still in its infancy in psychiatry, other medical disciplines have already evolved to a better understanding of its role and potential therapeutic implications. For instance, the role of microbiome alterations in a potentially lethal intestinal infection caused by Clostridium difficilis has been clearly established. In fact, it is now clear that Clostridium difficilis spreads and becomes dangerous only when antibiotics destroy the gut's ordinary bacterial residents that otherwise prevent it from overgrowing. Interestingly, when specific antibiotics against Clostridium difficilis fail, a fecal transplant from a healthy gut, able to provide bacteria able to suppress Clostridium difficilis may be highly effective (Brandt, 2012; Burke and Lamont, 2013; Gens et al., 2013). However, the efficacy of fecal transplantation for psychiatric disorders in general, and schizophrenia in particular, is yet to be appropriately tested, despite encouraging preliminary data. For instance, an open-label trial (Kang et al., 2017) demonstrated that children with autism treated with fecal transplantation showed improved behavior.

Clearly, more studies on the role of the microbiome, probiotics, prebiotics and fecal transplantation in schizophrenia in general, and its resistant forms in particular, are in order.

## Conclusions

We are still far from considering the possibility of microbiome targeted treatments in patients with mental illness in general, and with schizophrenia in particular. Nonetheless, TRS remains a challenge for researchers and practitioners and the possibility of a role of the microbiome and of immune-mediated pathology should be better explored, not only in animal models but also in clinical trials of agents that are able to alter gut microbiota and possibly influence the mechanisms of gastrointestinal inflammation. In fact, given that alterations in the immune system (e.g., interleukins mediated inflammatory processes and in T-cell functions), have been considered among the neurobiological correlates of treatment resistant schizophrenia (Altamura et al., 2005), the possibility of agents that are able to modulate such processes should be better explored. More studies on the relationship between schizophrenia and immune system alterations, as well as on the gut microbiome and on the possibility that agents such as, probiotics may contribute to the treatment of inflammatory processes within the gastrointestinal system, and exert positive effects on the symptoms of schizophrenia that are otherwise resistant to the classic medications, are warranted.

## Author contributions

All authors contributed to conceptualization, interpretation of reviewed papers, drafting and review of the present paper. AC, AG, and AF also selected the papers that were the object of this review.

### Conflict of interest statement

AF is/has been a consultant and/or a speaker and/or has received research grants from Allergan, Angelini, Generici DOC, Lundbeck, Italfarmaco, Janssen, Otsuka, Pfizer, Recordati, Roche, Sonofi Aventis, Vifor. The remaining authors declare that the research was conducted in the absence of any commercial or financial relationships that could be construed as a potential conflict of interest.
